# *Salmonella enterica* Serovars Typhi and Paratyphi A are avirulent in newborn and infant mice even when expressing virulence plasmid genes of *Salmonella* Typhimurium

**DOI:** 10.3855/jidc.1218

**Published:** 2010-11-24

**Authors:** M. Javier Santander, Roy Curtiss

**Affiliations:** The Biodesign Institute, Centre for Infectious Diseases and Vaccinology. Arizona State University, Tempe, Arizona

**Keywords:** *Salmonella* Typhi, *Salmonella* Paratyphi A, newborn mice, infant mice, virulence plasmid

## Abstract

**Background:**

Salmonella enterica serovars Typhi and Paratyphi A are human host-restricted pathogens. Therefore, there is no small susceptible animal host that can be used to assess the virulence and safety of vaccine strains derived from these Salmonella serovars. However, infant mice have been used to evaluate virulence and colonization by another human host-restricted pathogen, Vibrio cholerae.

**Methodology:**

The possibility that infant mice host could be adapted for Salmonella led us to investigate the susceptibility of newborn and infant mice to oral infection with S. Typhi and S. Paratyphi A. Salmonella enterica serovar Typhimurium causes enteric fever in adult mice and this system has been used as a model for human typhoid. The pSTV virulence plasmid, not present in S. Typhi and S. Paratyphi A, plays an essential role in S. Typhimurium colonization and systemic infection of mice. We also conjugated pSTV into S. Typhi and S. Paratyphi A serovars and evaluated these transconjugants in newborn and infant mice.

**Results:**

We determined that the spv virulence genes from the S. Typhimurium virulence plasmid are expressed in S. Typhi and S. Paratyphi A in a RpoS dependent fashion. Also, we determined that S. Typhi and S. Paratyphi A with and without pSTV transiently colonize newborn and infant mice tissues.

**Conclusion:**

Newborn and infant mice infected with S. Typhi and S. Paratyphi A do not succumb to the infection and that carriage of the S. Typhimurium virulence plasmid, pSTV, did not influence these results.

## Introduction

Typhoid and paratyphoid fever are severe human diseases caused by *S.* Typhi and *S*. Paratyphi A respectively, with an estimated 16 million cases resulting in more than 600,000 deaths annually [1,2]. Both *S.* Typhi and *S.* Paratyphi A are human host-restricted pathogens. The pathogenesis of typhoid and paratyphoid are poorly understood, in part due to the lack of a susceptible animal host that exhibits the same clinical signs as human infections. Attenuated *S.* Typhi strains have been used as live vectors to deliver foreign antigens either by expressing antigens or by delivering the antigen-encoding genes on eukaryotic expression plasmids [3,4,5]. The absence of an inexpensive, small animal host for pre-clinical evaluation of vaccine candidates is an obstacle to developing live attenuated *S.* Typhi vaccines. Chimpanzees infected with wild-type *S*. Typhi Ty2 produced a mild clinical illness that resembled human typhoid fever, but only when given in a high dose (1x10^11^ CFU) [6]. This host is not convenient for high through-put analyses due to the high cost and scarcity of supply. In addition, since a high inoculum of wild-type is needed to cause clinical infection, this is not an ideal host for evaluating the virulence potential of attenuated strains [7].

*S.* Typhi and *S.* Paratyphi A are unable to induce progressive disease in adult BALB/c mice challenged orally or parenterally with high doses ( > 10^9^ orally; 10^7^ to 10^8^ parenterally) [8,9,10,11,12]. The current method for assessing the safety of *S.* Typhi vaccines consists of inoculating mice intraperitoneally with moderated doses ( > 10^3^ CFU) of *S.* Typhi suspended in hog gastric mucin [7]. However, death of the mice is believed to result from the toxic effects of endotoxin associated with the rapidly expanding peritoneal population of *S*. Typhi [13,14,15]. In addition, the attenuating effects of some mutations cannot be discerned by this method [16]. The use of other animal hosts, such as rabbits and pigs, has also been explored, but these animals were found to be no more useful than mice [17, 18]. However, intranasal inoculation of mice has been used successfully to evaluate the immune responses to foreign antigens expressed by *S.* Typhi recombinant vaccines [19,20].

*S.* Typhimurium, the causative agent of enterocolitis infection in humans and cattle, causes a lethal systemic disease in susceptible mice that resembles human typhoid infection [21,22]. The mouse assay has been adopted and extensively used to study pathogenesis and immunity of typhoid fever. However, a shortcoming of this assay is the fact that *S.* Typhimurium does not cause typhoid fever in humans, suggesting that genetic differences between *S.* Typhi and *S.* Typhimurium are critically important for the disease outcome in both mice and humans. Whole-genome sequencing has revealed genome degradation in host-restricted *Salmonella* serotypes [23]. Therefore, the evolution from a broad host range serovar such as Typhimurium, to host-restricted serovars such as Typhi and Paratyphi A, may have occurred by genome degradation [21,23]. In addition, not all the information obtained using the *S*. Typhimurium mouse assay can be directly applied to improve understanding of typhoid fever since some of the virulence factors of *S.* Typhimurium such as *Salmonella* virulence plasmid pSTV, required for invasion of host tissues [24,25], are absent in *S*. Typhi and *S*. Paratyphi A [23,26,27,28].

Nevertheless, infant mice have been used to measure the median lethal dose (LD_50_) as a parameter for disease production by *Vibrio cholerae* and other host-restricted bacterial strains [29,30,31] and therefore may be useful in developing a systemic infection model for *S.* Typhi and *S.* Paratyphi A, since adult mice are resistant to those pathogens [10]. In this study, the colonization and pathogenesis potential of *S.* Typhi and *S*. Paratyphi A in newborn and infant mice was evaluated. Newborn and infant mice were observed to be colonized by *S.* Typhi and *S.* Paratyphi A, but were tolerant of the infection. In addition, whether *S*. Typhi and *S.* Paratyphi A carrying and expressing the *S.* Typhimurium virulence plasmid were able to better infect and colonize newborn and infant mice was evaluated.

## Methodology

### Bacterial strains and culture conditions

The bacterial strains and plasmids used in this study are listed in Table 1. Bacteriological media and components were from Difco (Franklin Lakes, NJ). Antibiotics and reagents were from Sigma (St. Louis, MO). *Salmonella* strains were grown at 37°C in either buffered magnesium minimal medium pH 5.5 (MgM) [32] or LB medium [33]. For plates, media was solidified with 1.5% (wt/vol) agar. When required, medium was supplemented with tetracycline (tet; 12.5 μg/ml), L-cysteine-HCl (cys; 22 μg/ml), DL-tryptophan (trp; 20 μg/ml) and L-histidine-HCl (his; 22 μg/ml). Buffered saline with gelatin (BSG) [34] was used as a diluent and to suspend bacteria prior to inoculation of mice.

### Beta-galactosidase assays

Expression of *spvR-lacZ* and *spvA-lacZ* fusions in RpoS^+^ and RpoS^−^*S*. Typhi was determined by β-galactosidase activity assay [35]. Strains were transformed with pGTR72 (*spvR-lacZ*, operon fusion), pGTR90 (*spvA-lacZ*, operon fusion) or pGTR75 (*tet-lacZ* operon fusion control) [36] and grown in MgM medium to stationary phase. The *spv* operon is up-regulated under conditions that mimic the *Salmonella* containing vacuole (SCV) [33]. MgM media was used for this experiment because it mimics the environment of SCV [37].

### Construction and characterization of *S*. Typhi and *S*. Paratyphi A carry pSTV

*S* Typhi and *S*. Paratyphi A strains harboring the *S.* Typhimurium virulence plasmid were constructed by conjugation on minimal media [38]. Plasmid pStSR101 is a Tn*mini-tet-*labeled virulence plasmid derivative, which can restore the full virulence of pSTV-cured strains of *S.* Typhimurium [25]. Like the wild-type virulence plasmid, it is self-transmissible [38]. *S.* Typhimurium χ3351 SL1344 *hisG* carrying pStSR101 was used as the donor. Conjugation was performed in M9 minimal media agar [39], supplemented with cys, trp, and his. Selection was performed on M9 media supplemented with cys, trp and tet to select for *S.* Typhi and *S.* Paratyphi A transconjugants and against the histidine-requiring *S.* Typhimurium donor. Transconjugants were characterized for LPS, Vi antigen (*S*. Typhi), biochemical properties, and nutritional requirements as described [39] and were found to exhibit the expected phenotypes (Table 1). The stability of pStSR101 in *S*. Typhi and *S*. Paratyphi A was determined essentially as described by Konjufca *et al*. [40], except that strains were grown in the absence of tetracycline for fifty generations, at which point cells were plated onto LB agar and individual colonies were screened for tetracycline resistance.

### Reverse Transcriptase-PCR

Expression of *spvR* and *spvA* in *S*. Typhi and *S*. Paratyphi A from pStSR101 was evaluated by RT-PCR. Total RNA extraction was performed by RNeasy QIAgene kit (Hilden, Germany) from strains grown in MgM media at 37°C to stationary phase [33,37]. Reverse transcription and PCR was performed using the one-step RT-PCR QIAgene kit. Specific primers were used for the *spvR* (5′-GGAAACAGGTTCCTTCAGTATCGC-3′ and 5′-TATTTGGCTGTTAACGGCTCTCCC-3′) (size of the *spvR* amplified fragment: 349 bp) and *spvA* (5′-TTGTCCGTCAGACCCGTAAACAGT-3′ and 5′-TCTTCCAGCGACACATCGGTATT CAG-3′) (size of the *spvA* amplified fragment: 358 bp) genes. *16S rRNA* primers were used as control of expression (5′-ACTGGCAGGCTTGAGCTTGTAGA-3′ and 5′-AAGGGCACAACCTCCAAGTA GACA-3′) (size of the *16S rRNA* amplified fragment: 158 bp).

### Animal experiments

BALB/c newborn and infant mice (Charles River Laboratories, Wilmington, MA) were bred and maintained at 22°C to 23°C with 12 hours of illumination daily. Mice older than two weeks were separated from their mothers 4 hours before infection and fed with regular food. Bacterial strains were grown overnight in standing cultures that were diluted 1:100 in prewarmed LB broth and grown with mild aeration to an OD_600_ of 0.8 to 0.9. Bacteria were sedimented by centrifugation at room temperature and resuspended in BSG to densities appropriate for the inoculation route and dose. Newborn mice (3 to 24 hours) and infant mice (48 hours to 3 weeks old) were challenged with ~10^9^ CFU of *S.* Typhi, *S.* Paratyphi A, or *S.* Typhimurium. Ten microliters of ~10^9^ CFU of the bacterial strain suspended in BSG were orally administered. Intranasal inoculations consisted in 5 μl of ~10^9^ CFU of the bacterial strain suspended in BSG, administered without anesthesia. Mice were euthanized via asphyxiation with CO_2_ and necropsied at various times. The bacterial titers in newborn mice inoculated orally were determined at 3, 7, 14 and 21 days post oral infection. Spleen, liver, and the intestines were collected and washed with BSG until homogenization. The homogenizer (Brinkman, Westburg, NY) was washed with 5% Amphyl, followed by a wash with 70% ethanol, followed by two washes with dH2O. Homogenized tissues were plated onto MacConkey agar plates supplemented with 1% lactose to determine the number of viable bacteria. *Salmonella* colonies were white on the MacConkey plates. Isolated colonies were further identified by agglutination with *Salmonella* specific antiserum (Table 1) and antibiotic resistance marker.

### Statistics

Mann-Whitney U Test (version 5.0; GraphPad Software, Inc.) was used for comparing the expression of *spv-lacZ* fusions.

## Results

### Virulence and colonization of *S.* Typhi and *S.* Paratyphi A in newborn and infant mice

**Figure 1:**
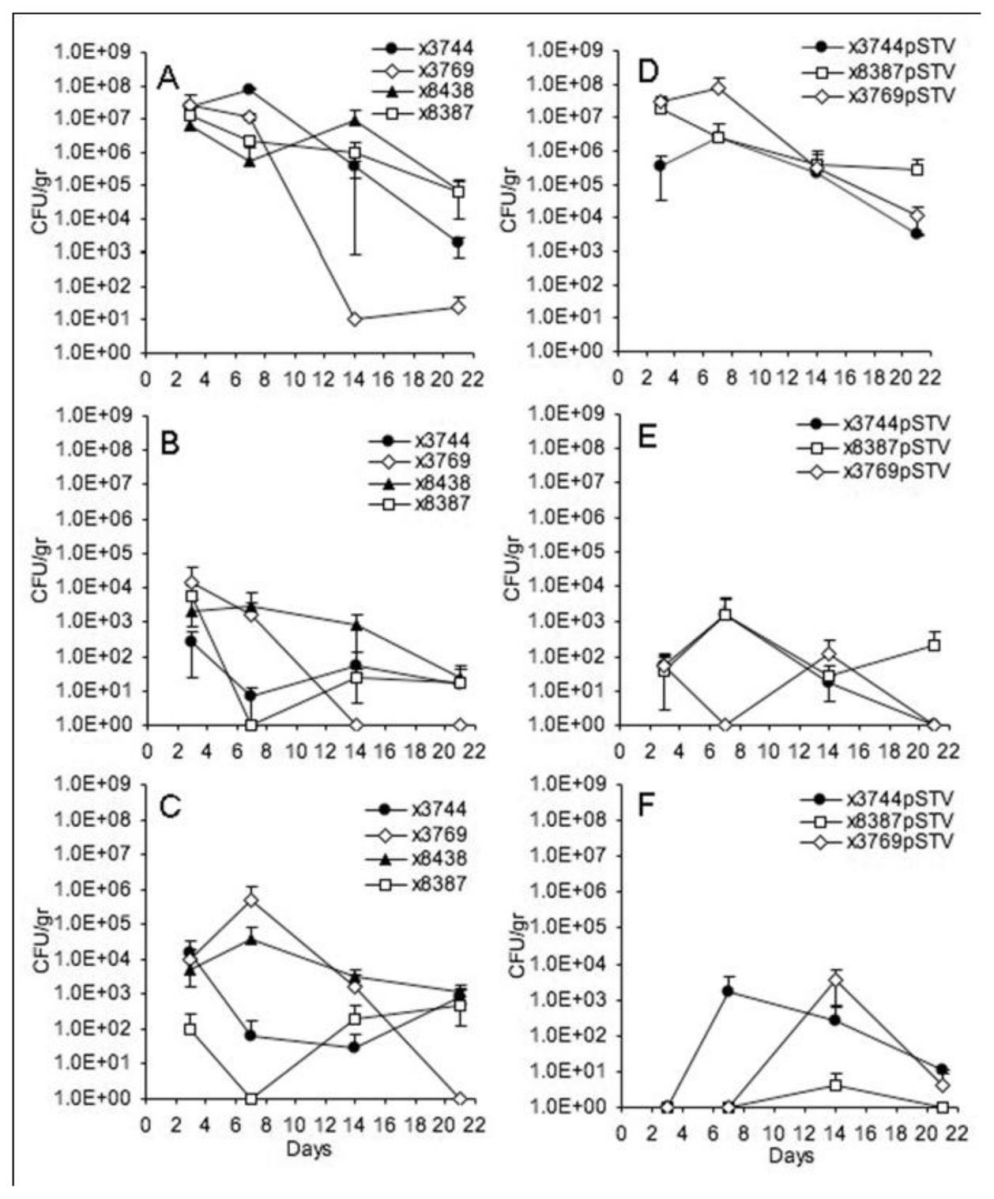
Colonization of *S.* Typhi and *S.* Paratyphi A with and without pStSR101 virulence plasmid in newborn mice < 24 h old. **A–C.** Newborn mice orally infected with *Salmonella* without pStSR101; **D–F.** Newborn mice orally infected with *Salmonella* harboring pStSR101; **A, D**. Intestine colonization; **B, E**. Spleen colonization; **C, F.** Liver colonization. Each point represents the average between 4 animal tissues.

Newborn and infant mice infected orally with *S*. Typhi and *S*. Paratyphi A survived without any symptoms of disease, while the mice inoculated with a *S.* Typhimurium strain cured of plasmid pSTV succumbed to the infection (Table 2). Infant mice, one to three weeks of age, and newborn mice infected intranasally with *S*. Typhi and *S*. Paratyphi A strains also survived without any symptoms of disease (Table 2). The bacterial titers in newborn mice inoculated orally were determined at 3, 7, 14 and 21 days post infection. Spleen, liver, and the intestines were collected and the number of viable bacteria was determined. *S.* Typhi RpoS^+^ and *S.* Paratyphi A RpoS^+^ were able to colonize the intestines for three weeks (Figure 1A). *S.* Typhi Ty2 RpoS^−^ was less able to persist in the intestines and cleared after one week. *S*. Typhi and *S.* Paratyphi A were able to colonize the spleen and liver of infected mice and the RpoS- strain Ty2 was more effectively cleared than the RpoS^+^ strains (Figures 1B and 1C). These results are consistent with a report that *rpoS S.* Typhimurium mutants are less persistent in mice than their wild-type RpoS^+^ parent strains [41, 45]. Taken together, these results show that although wild-type *S.* Typhi and *S.* Paratyphi A can transiently colonize young mice, they are not capable of establishing a disseminating infection.

### Expression of spv genes in *S.* Typhi RpoS^+^ and RpoS

The *S.* Typhimurium virulence plasmid is required for colonization of mouse tissues [24]. An investigation into whether the addition of the virulence plasmid to *S.* Typhi and *S*. Paratyphi A could enhance their ability to colonize young mice was conducted. One virulence plasmid operon that is critical for host invasion is encoded in the *spv* region [24,33,46]. The *spv* region consists of five genes, *spvRABCD* which are all transcribed in the same direction [46]. The *spvR* gene encodes SpvR, which activates the transcription of both the *spvR* and *spvABCD* transcriptional units [12,47,48]. In addition, the sigma factor RpoS is also required for maximum expression of the operon [49,50,51,52].

**Figure 2:**
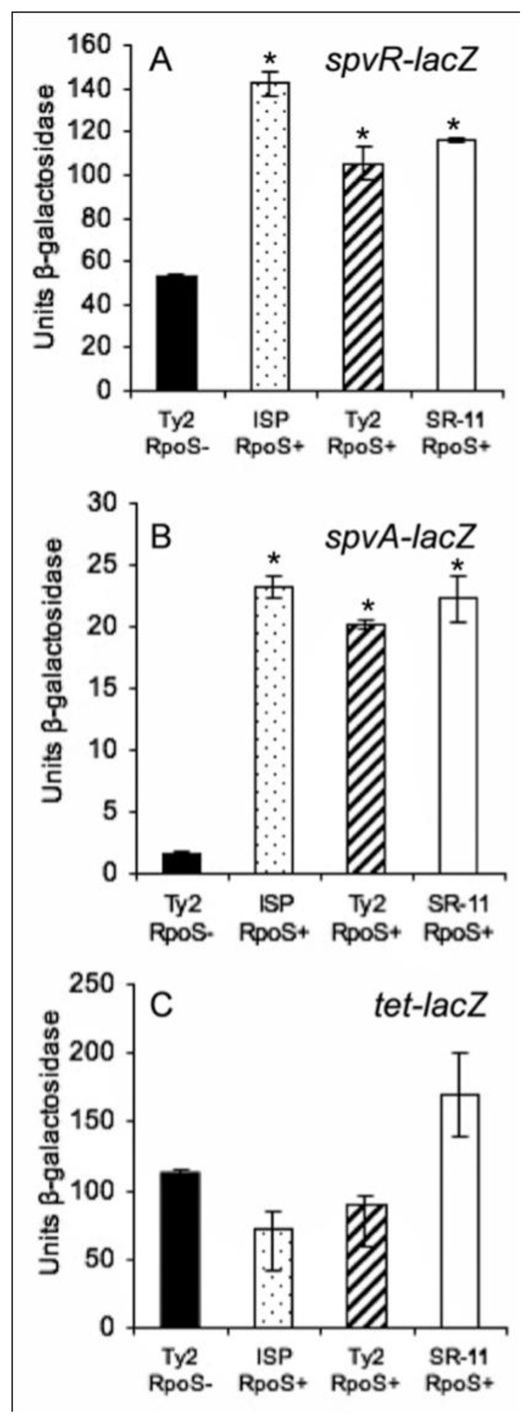
Evaluation of *spvR* and *spvA* expression in *S.* Typhi by β-galactosidase assay. **A.** Evaluation of *spvR*; **B.** Evaluation of *spvA*; **C.** Control. χ3769 *S.* Typhi Ty2 RpoS^−^; χ3744 *S.* Typhi ISP1820 RpoS^+^; χ3337 *S.* Typhimurium SR-11 RpoS^+^; strains harboring the respective plasmids; the strains where grown in MgM media to stationary growth phase. *P<0.01 for the RpoS^+^ strains compared with RpoS^−^ strain, significant differences are indicated.

Because of the central role of *spv* in tissue colonization, expression of *spvR-lacZ* and *spvA-lacZ* fusions in RpoS^+^ and RpoS^−^ *S*. Typhi by β-galactosidase activity were compared to establish that these genes were able to be transcribed in *S.* Typhi and that transcription is dependent upon RpoS. [35]. The RpoS^+^ *S.* Typhi strains produced β-galactosidase levels comparable to the *S.* Typhimurium RpoS^+^ control for both fusions (Figures 2A and 2B). In contrast, reduced expression was observed for *S.* Typhi Ty2, which has a defective *rpoS* allele due to a frame-shift mutation at nucleotide 993 [53]. These results indicate that *S*. Typhi is able to transcribe the *spv* genes to the same levels as *S.* Typhimurium and confirm that maximum *spv* expression requires RpoS.

### Stability and expression of pSTV plasmid in *S.* Typhi and *S.* Paratyphi A

**Figure 3:**
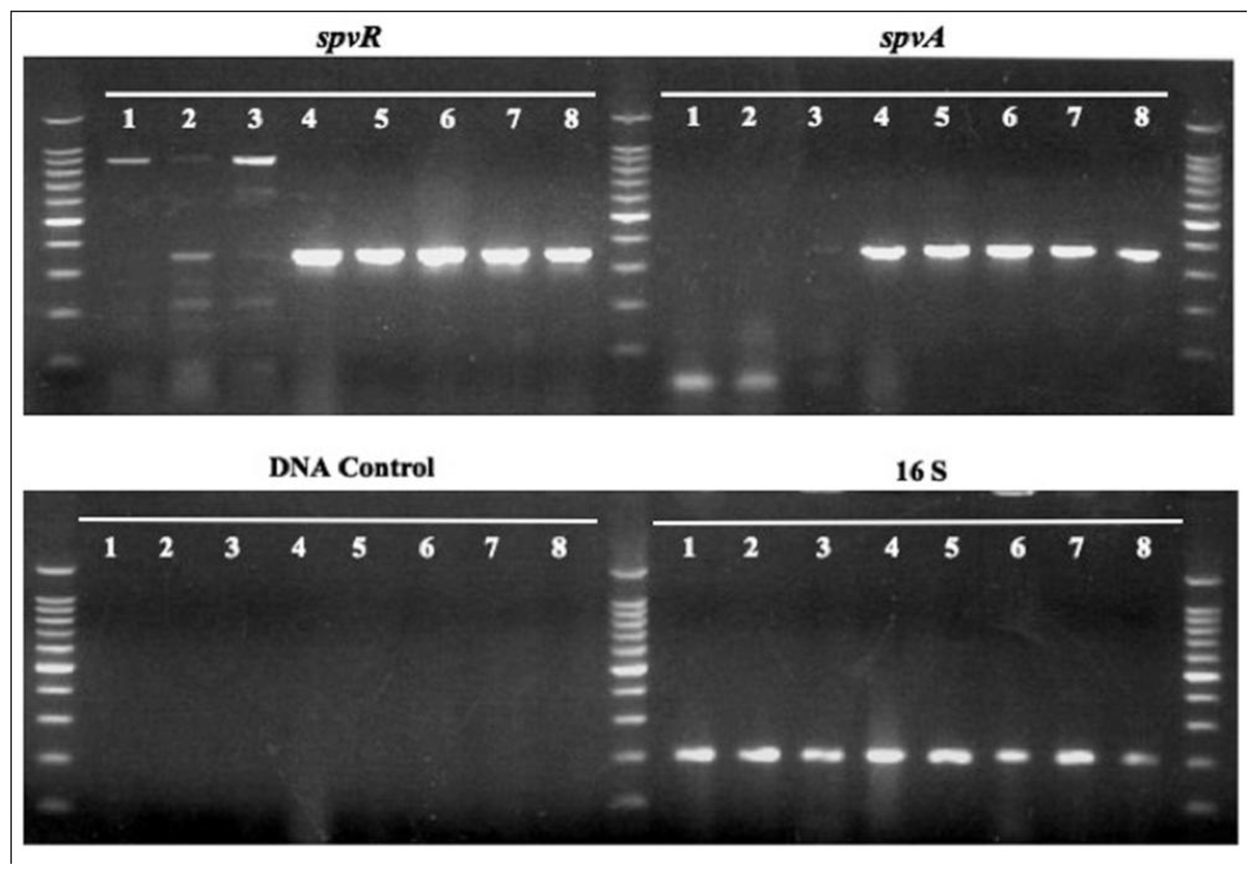
Expression of *spvR* and *spvA* evaluated by RT-PCR (agarose gel 1%). **1.** χ3769 *S.* Typhi Ty2; **2.** χ3744 *S.* Typhi ISP1820; **3.** χ8387 *S.* Paratyphi A; **4.** χ3769 *S.* Typhi Ty2 (pStSR101); **5**. χ3744 *S.* Typhi ISP1820 (pStSR101); **6.** χ8387 *S.* Paratyphi A (pStSR101); **7.** χ3351 *S.* Typhimurium SL-1344 (pStSR101); **8.** χ3761 *S.* Typhimurium UK-1 pSTV^+^; **16S**: 16S (*rrnA*) was used as positive control; **DNA control**: the RNA samples were used as templates in a PCR reaction to amplify the *16SrRNA* to detect DNA contamination.

A tetracycline-marked derivative of the *S.* Typhimurium virulence plasmid, pStSR101 [24], was moved into the *S.* Typhi and *S.* Paratyphi A strains. Because these host-restricted strains do not normally carry this plasmid, it is possible that the plasmid may not be maintained for enough generations to colonize a mouse. Therefore, the stability of pStSR101 in *S*. Typhi and *S*. Paratyphi A was examined. The pStSR101 virulence plasmid was stably maintained for 50 or more generations in all *S.* Typhi and *S.* Paratyphi A strains. In addition, whether the *spv* genes were transcribed from this plasmid was evaluated. RT-PCR products were detected in all strains harboring pStSR101 (Figure 3), indicating that the *spv* genes are transcribed in *S.* Typhi and *S.* Paratyphi A.

### Virulence and colonization of *S*. Typhi and *S.* Paratyphi A harboring pSRSt101 in newborn and infant mice

The effect of the *S.* Typhimurium virulence plasmid on the ability of *S*. Typhi and *S*. Paratyphi A to cause disease in newborn and infant mice was evaluated. When newborn and infant mice were orally inoculated with *S.* Typhi and *S.* Paratyphi A harboring pStSR101, they survived without any disease symptoms, whereas all the mice inoculated with *S.* Typhimurium died (Table 3). Infant mice and newborn mice infected intranasally with *S.* Typhi or *S.* Paratyphi A carrying pStSR101 also survived without any symptoms of disease (Table 3).

The presence of pStSR101 did not enhance the ability of *S.* Typhi and *S.* Paratyphi A to colonize infant mice (Figure 1). Unexpectedly, the presence of pStSR101 in *S.* Typhi Ty2 RpoS^−^ resulted in a slight increase in persistence in the intestines compared to the plasmid-free strain (Figures 1A and 1D), indicating that there may be a virulence plasmid gene(s) that can complement the defect in intestinal colonization imparted by the RpoS^−^ phenotype. Recently, it has been reported that pP_ST98_, a promiscuous R plasmid found in a multi-drug resistant isolate of *S.* Typhi from Asia, carries the *spv* region. This plasmid confers antibiotic resistance and increases virulence in mice when transferred to pSTV^−^ *S.* Typhimurium [54]. While it is possible that *spv* genes may confer an enhancement of virulence in human hosts, our results indicate that they have no effect on infant mouse colonization in wild-type *S.* Typhi.

## Discussion

In summary, these results show that *S*. Typhi and *S*. Paratyphi A can transiently colonize young mice, but cannot establish a lethal infection. The *spv* genes are transcribed in *S*. Typhi and *S*. Paratyphi A and a functional *rpoS* gene is required for maximum expression. However, the virulence plasmid does not enable *S*. Typhi or *S*. Paratyphi A to establish a lethal infection in newborn or infant mice.

## Figures and Tables

**Table 1:** *Salmonella* strains and relevant characteristics

Strain	Relevant characteristics	Origin	Source or reference
χ3744 *S.* Typhi ISP1820	Wild-type, RpoS^+^, Cys^−^, Trp^−^, OD_1_:H_d_:-:Vi, V & V/W form	Wild-type	41
χ3744 *S.* Typhi ISP1820 (pStSR101)	Wild-type RpoS^+^, Cys^−^, Trp^−^, OD_1_:H_d_:-:Vi, V & V/W form, Tet^r^	χ3744	This study
χ3761 *S*. Typhimurium UK-1	RpoS^+^, pSTV, OB_1_:H_i_:H_2_:-	Wild-type	42
χ3769 *S.* Typhi Ty2	Wild-type, RpoS^−^, Cys^−^, OD_1_:H_d_:-:Vi, V form	Wild-type	43
χ3769 *S.* Typhi Ty2 (pStSR101)	Wild-type, RpoS^−^, Cys^−^, OD_1_:H_d_:-:Vi, V form, Tet^r^	χ3769	This study
χ8219 *S.* Paratyphi A	RpoS^+^, Cryptic plasmid pSPA1, OA:H_a_:-:-	Wild-type	ATCC 9281
χ8387 *S.* Paratyphi A	RpoS^+^, Cryptic plasmid cured, OA:H_a_:-:-	χ8219	This study
χ8387 *S.* Paratyphi A (pStSR101)	RpoS^+^, OA:H_a_:-:-, Tet^r^	χ8387	This study
χ8438 *S.* Typhi Ty2	RpoS^+^, Cys^−^, OD_1_:H_d_:-:Vi, V & V/W form	χ3769	41
χ8438 *S.* Typhi Ty2 (pStSR101)	RpoS^+^, Cys^−^, OD_1_:H_d_:-:Vi, V & V/W form, Tet^r^	χ8438	This study
χ8740 *S*. Typhi CT18	RpoS^+^, Vi^−^, OD_1_:H_d_:-:-, W form	Wild-type	27
χ3337 *S*. Typhimurium SR-11	RpoS^+^, *gyrA1816*, pSTV^−^, OB_1_:H_i_:H_2_:-	χ3306	25
χ3351 *S*. Typhimurium SL-1344 (pStSR101)	RpoS^+^, *rpsL*, *hisG*, Tet^r^, OB_1_:H_i_:H_2_:-	χ3340	25
**Plasmids**			
pGTR72	*spvR-lacZ,cat*		44
pGTR90	*spvA-lacZ,cat*		44
pGTR75	*spvR::lacZ*,*cat* cat, same as pGTR72, except the *lacZ*,*cat* is inserted in opposite orientation, under control of *tet* promotor of the plasmid		44
pSTV	*spvRABCD*, *pefBACD*, *rck*		25
pStSR101	*spvRABCD*, *pefBACD*, *rck*, Tn*mini-tet-*		25

**Table 2:** Infection of newborn and infant mice with *S.* Typhi and *S.* Paratyphi A.

Strain	Inoculating dose (CFU)	Mice age	Survivors/total	Route
χ3744 *S.* Typhi ISP1820	1.1 x 10^9^	3 weeks	5/5	oral
	1.1 x 10^9^	1 week	7/7	oral
	1.1 x 10^9^	<24 h	6/6	oral
	1.1 x 10^9^	1 week	7/7	intranasal
χ3769 *S.* Typhi Ty2 (RpoS^−^)	1.1 x 10^9^	<24 h	5/5	oral
χ8438 *S.* Typhi Ty2 (RpoS^+^)	1.5 x 10^9^	<24 h	4/4	oral
χ8740 *S.* Typhi CT18	1.0 x 10^9^	<24 h	8/9	oral
χ8387*S.* Paratyphi A	1.1 x 10^9^	<24 h	8/8	oral
	1.1 x 10^9^	1 week	9/9	intranasal
χ3337 *S*. Typhimurium SR-11	2.1 x 10^9^	3 weeks	0/5	oral
	2.1 x 10^9^	<24 h	0/5	oral

Newborn: 3 h to 24 h after birth. Infant: 48 h to 3 weeks old. Mice were observed for 4–5 weeks.

χ3337 *S.* Typhimurium pSTV-cured was used as positive control.

**Table 3:** Infection of newborn and infant mice with *S.* Typhi pStSR101 and *S.* Paratyphi A pStSR101.

Strain	Inoculating dose (CFU)	Mice age	Survivors/total	Route
χ3744 *S.* Typhi ISP1820 (pStSR101)	1.2 x 10^9^	<24 h	6/6	oral
	1.1 x 10^9^	<24 h	7/7	intranasal
χ3769 *S.* Typhi Ty2 (pStSR101)	1.2 x 10^9^	<24 h	5/5	oral
χ8387 *S.* Paratyphi A (pStSR101)	1.1 x 10^9^	<24 h	10/10	oral
	1.1 x 10^9^	<24 h	7/7	intranasal
χ3351 *S.* Typhimurium SL-1344 (pStSR101)	1.8 x 10^9^	3 weeks	0/5	oral
	1.8 x 10^9^	<24 h	0/8	oral

Newborn: 3 h to 24 h after birth. Infant: 48 h to 3 weeks old. Mice were observed between 4–5 weeks.

χ3351 *S.* Typhimurium (pStSR101) was used as positive control.
